# Innate immunity in ocular *Chlamydia trachomatis *infection: contribution of IL8 and CSF2 gene variants to risk of trachomatous scarring in Gambians

**DOI:** 10.1186/1471-2350-10-138

**Published:** 2009-12-16

**Authors:** Angels Natividad, Jeremy Hull, Gaia Luoni, Martin Holland, Kirk Rockett, Hassan Joof, Matthew Burton, David Mabey, Dominic Kwiatkowski, Robin Bailey

**Affiliations:** 1 London School of Hygiene and Tropical Medicine, London University, London, UK; 2 Wellcome Trust Centre for Human Genetics, University of Oxford, Oxford, UK; 3 Medical Research Council Laboratories, Fajara, The Gambia; 4 Laboratoire de Génétique Humaine des Maladies Infectieuses - INSERM U550, Faculté de Médecine Necker, 156 rue de Vaugirard, 75015 Paris, France

## Abstract

**Background:**

Trachoma, a chronic keratoconjunctivitis caused by *Chlamydia trachomatis*, is the world's commonest infectious cause of blindness. Blindness is due to progressive scarring of the conjunctiva (trachomatous scarring) leading to in-turning of eyelashes (trichiasis) and corneal opacification. We evaluated the contribution of genetic variation across the chemokine and cytokine clusters in chromosomes 4q and 5q31 respectively to risk of scarring trachoma and trichiasis in a large case-control association study in a Gambian population.

**Methods:**

Linkage disequilibrium (LD) mapping was used to investigate risk effects across the 4q and 5q31 cytokine clusters in relation to the risk of scarring sequelae of ocular *Ct *infection. Disease association and epistatic effects were assessed in a population based study of 651 case-control pairs by conditional logistic regression (CLR) analyses.

**Results:**

LD mapping suggested that genetic effects on risk within these regions mapped to the pro-inflammatory innate immune genes interleukin 8 (IL8) and granulocyte-macrophage colony stimulatory factor (CSF2) loci. The IL8-251 rare allele (IL8-251 TT) was associated with protection from scarring trachoma (OR = 0.29 p = 0.027). The intronic CSF2_27348 A allele in chromosome 5q31 was associated with dose dependent protection from trichiasis, with each copy of the allele reducing risk by 37% (p = 0.005). There was evidence of epistasis, with effects at IL8 and CSF2 loci interacting with those previously reported at the MMP9 locus, a gene acting downstream to IL8 and CSF2 in the inflammatory cascade.

**Conclusion:**

innate immune response SNP-haplotypes are linked to ocular *Ct *sequelae. This work illustrates the first example of epistatic effects of two genes on trachoma.

## Background

Trachoma is the leading infectious cause of blindness. Eighty-five million children have active (inflammatory) trachoma, and about 7 million people, mainly adults, are blind from late scarring sequelae [1]. For demographic reasons, the number of people blind due to trachoma may be increasing [2]. In trachoma, ocular *Chlamydia trachomatis (Ct) *infection causes inflammatory changes in the conjunctiva, and repeated infections sometimes lead to fibrosis and scarring of the sub tarsal conjunctiva. This may cause the upper eyelid margin to turn inwards so that the lashes rub against the eyeball (trichiasis), which damages the cornea and leads ultimately to blindness [3].

In trachoma endemic areas most individuals suffer from ocular *Ct *infection in childhood. The majority resolve the infection without permanent sequelae. However, some individuals develop severe and persistent clinical disease in response to infection [4,5], and are more likely to develop conjunctival scarring and trichiasis in later life [6]. The reasons for this heterogeneity in susceptibility to chlamydial infection and disease progression following a rather uniform bacterial exposure remain incompletely understood; however the host genetic background may account for part of the risk. Some susceptibility loci have been mapped to cytokine genes [7-13], but most of the heritable risk effects in scarring trachoma and trichiasis are uncharacterised. Identification of genetic loci which contribute to the burden of trachoma sequelae in the population may help to dissect the immunopathology of ocular *Ct *infection in humans.

It has been postulated that the early secretion of pro-inflammatory cytokines and chemokines by epithelial cells following *Ct *infection may initiate and sustain a chronic inflammatory process associated with pathology [14-16]. The chromosomal regions 4q and 5q31, which are rich in chemokine and cytokine loci respectively, have been implicated in susceptibility to several common diseases [17-19]. They contain the genes interleukin 8 (IL8) and granulocyte-macrophage colony stimulatory factor (CSF2) which have strong candidacy to affect risk of the scarring complications of trachoma. However, studies of diversity in these chromosomal segments in the Gambian population revealed complex genetic features, including low haplotypic diversity of IL8 [20,21] and a long-range linkage disequilibrium (LD) pattern spanning CSF2 [22], both of which extend for several hundred kilobases. Therefore, observed disease association at the IL8 and CSF2 loci may potentially map to one or more of the neighbouring genes. We report here an analysis of the risk effects at IL8 and CSF2, in a Gambian case-control association study of scarring trachoma and trichiasis, which took the LD structure at these loci into account.

## Methods

### Study population

651 subjects with scarring trachoma, identified by clinical examination using WHO criteria and unrelated controls with normal eyelids pair-matched by age, sex, ethnicity and village of residence, were recruited for the study. These were genotyped in two phases; in the first phase, 373 case-control pairs were typed using the full marker sets. 269 of the cases had trichiasis in addition to scarring. Mean age was 39 years (range 5-90), and 273 pairs were female (73%). 32% were of Mandinka ethnicity, 30% Wolof and 25% Jola. In the second phase, 278 new case-control samples were further typed with the reduced marker set, 38 cases had trichiasis in addition to scarring. Mean age was 34 years, and 193 pairs were female (68%). 5% were Mandinka, 56% Wollof, 24% Fula and 9% Jola. The sample populations of the two case-control phase studies (phase 1+ phase 2) differed slightly in age group and ethnic composition. In the pooled dataset, 651 Gambian subjects with scarring trachoma (307 of them also had trichiasis), and pair-matched controls with normal eyelids, 268 individuals were Mandinka (25.8%), 542 were Wolof (33.3%), 236 were Jola (22.6%), 164 were Fula(10.8%) and 90 (7.5%) were of other ethnic origin. 69% of the recruited subjects were female and the mean age of the population under study was 37 years.

Subjects gave written consent. The study and its procedures were approved by the Gambia Government/MRC Ethics Committee (SCC 729/857), the Ethics committees of the London School of Hygiene and Tropical Medicine and of Oxford University, and are in accordance with the Declaration of Helsinki. Subjects diagnosed with trichiasis were offered free corrective surgery.

### DNA extraction and SNP genotyping

Genomic DNA (gDNA) was isolated from either venous blood in EDTA or buccal brush samples. Genotypes were determined by the Sequenom system using matrix assisted laser desorption/ionization time-of-flight (MALDI-TOF) mass spectrometry as previously described [23]. Primer sequences are available on request.

### SNP selection

Initially 9 haplotype tagging SNPs (htSNPs) spanning a region 330 kb long across the IL8 locus in the Gambian population [21] (Table 1) and 16 informative htSNP markers capturing the haplotype block structure within a 656 kb segment of the 5q31 region in Gambians [22] (Table 2) were genotyped on a subset of 373 case-control pairs. From these marker sets, two reduced sets of 4 htSNPs covering the IL8 and CSF2 loci respectively were typed on the remaining 278 case-control pairs.

**Table 1:** Haplotype tagging SNPs on chromosome 4q genotyped in 373 Gambian case-control pairs

SNP position in haplotype	SNP id^a^	Chromosome position	Database id	SNP genomic location	COMMON allele (minor allele)	Allele frequency
1	AFP+8865	74300068	rs2298839	intron/exon	A(g)	0.41
2	**AFM+1666**	74338382	rs1894292	intron	A(g)	0.24
3	**AFM+4530**	74341246	rs1894293	intron	G(a)	0.33
4	**AFM+15790**	74352506	rs1158101	intron/exon	C(t)	0.14
5	**IL8-251**	74595248	rs4073	promoter	A(T)	0.14
6	IL8+396	74595893	rs2227307	intron	T(g)	0.47
7	IL8+37674	74633165	rs13109146	flanking	C(t)	0.04
8	IL8+39739	74635230	rs39739	flanking	G(a)	0.04
9	IL8+40050	74635538	rs2224434	flanking	C(a)	0.14

^a ^SNPs given in bold were selected for typing in the complete sample set of 651 case-control pairs.

**Table 2:** AFM/IL8 haplotype frequency estimates in cases and controls and risk estimates from CLR analysis of matched case-control pairs.

Haplotype a	# controls	freq	# cases(TS)	freq	p-value	CLR:OR (95%CI)	# controls	freq	# cases(TT)	freq	p-value	CLR:OR (95%CI)
AGGTAGTAC	215	0.35	195	0.34	-	reference	162	0.35	152	0.36	-	reference
GAATATTAC	79	0.13	108	0.19	0.061	1.42 (0.98, 2.04)	55	0.12	75	0.18	0.150	1.38 (0.89, 2.13)
AGGTATTAC	64	0.10	38	0.07	0.037	0.59 (0.36, 0.97)	49	0.11	32	0.08	0.113	0.62 (0.35, 1.12)
GAATAGTAC	50	0.08	51	0.09	0.453	1.20 (0.75, 1.90)	38	0.08	35	0.08	0.553	1.19 (0.68, 2.08)
GGGTAGTAC	50	0.08	42	0.07	0.829	0.94 (0.57, 1.58)	33	0.07	28	0.07	0.489	0.79 (0.41, 1.53)
GGATATTAC	39	0.06	23	0.04	0.127	0.62 (0.34, 1.15)	26	0.06	20	0.05	0.420	0.75 (0.38, 1.50)
AGGTTTTAA	33	0.05	23	0.04	0.323	0.74 (0.40, 1.35)	28	0.06	16	0.04	0.067	0.50 (0.24, 1.05)
AGGCAGTAC	23	0.04	26	0.05	0.597	1.18 (0.63, 2.22)	19	0.04	20	0.05	0.984	1.01 (0.49, 2.06)
GGACAGTAC	26	0.04	17	0.03	0.356	0.73 (0.38, 1.42)	20	0.04	13	0.03	0.469	0.75 (0.35, 1.62)
AGGCATTAC	26	0.04	30	0.05	0.229	1.48 (0.78, 2.80)	17	0.04	22	0.05	0.286	1.50 (0.71, 3.15)
GAATTTTAA	14	0.02	18	0.03	0.413	1.39 (0.63, 3.03)	12	0.03	10	0.02	0.892	0.93 (0.35, 2.49)
others	145	0.23	157	0.27	-	-	97	0.21	115	0.27	-	-

^a ^Haplotype configuration: AFP+8865, AFM+1666, AFM+4530, AFM+15790, IL8-251, IL8+396, IL8+37674, IL8+39739, IL8+40050. Others refers to population haplotypes of <10% frequency.

### Analytical methods

#### Allele and genotype frequencies

Estimates of allele and genotype frequencies were determined by dividing the total number of each observed allele (or genotype) by the total number of chromosomes in the population sample. Genotype distributions at all loci were in Hardy-Weinberg equilibrium (HWE) in both cases and controls.

#### LD estimates

The program HaploXT http://www.sph.umich.edu/csg/abecasis/gold/docs/haploxt.html was used to calculate pair-wise LD estimates for the markers, expressed as the abs D' and R2 parameters (the latter being independent of the allele frequencies in the population). The input file for HaploXT was the haplotypes for each control subject generated by PHASE v2 [24]. The HaploXT output file was used as an input for MARKER v1.0, a graphical output program written by D. Kwiatkowski at the Wellcome Trust Centre for Human Genetics (WTCHG, Oxford) http://gmap.net/. These estimates were used to construct haplotype blocks of high LD (and reduced within block diversity) within the regions studied.

#### Haplotype construction

The program PHASE v2 was used to infer haplotypes from population genotype data [25] and to estimate the frequency of each inferred haplotype for cases and controls. To check the accuracy of haplotype reconstruction PHASE inferred haplotypes were compared with those from the SNPHAP program for haplotype construction from population data http://www-gene.cimr.cam.ac.uk/clayton/software/snphap.txt.

#### Association analysis

An adjusted univariate analysis was first carried out by using Mantel-Haenszel (M-H) chi-square statistics (or Fisher's exact test if appropriate) to test for differences in minor allele, genotype and haplotype frequencies between cases of trachomatous scarring (TS) and of trichiasis (TT) with their pair-matched unrelated controls. Subsequently conditional logistic regression (CLR) analysis for disease association, taking into account matching of case-control pairs, was performed. In the presentation of results, reference genotypes were generally selected to be those that were present at the highest frequency in our study population. Genotypes conferring susceptibility or protection are accordingly represented by odds ratios (OR) of greater than or less than one respectively. A test for trend in the odds ratios was carried out to check for a dose response effect relationship between genotype/haplotype and disease allowing for potential confounders. All analysis was performed using STATA (v8.0) software.

#### Risk localisation analysis

To localise observed risk effects within the inferred population risk haplotype, PHASE assignments from a total of 764 population chromosomes from unrelated Gambian individuals were made for differing DNA segments by progressively reducing the number of nearby SNPs used to infer the population haplotypes. Association analyses were repeated each time until the risk effects (as measured by ORs) associated with each DNA segment delimited by those SNPs started to decrease.

#### Epistasis

The simplest system of two interacting loci using conditional logistic regression was used to assess epistasis between MMP9Q279R genotypes, which we previously reported as conferring risk to scarring trachoma and trichiasis, and genotypes at the unlinked IL8 and CSF2 loci. Because the combination analysis leads to small cell sizes, we also tested for allelic epistasis, whereby joint risk effects of two alleles were tested for deviation from additivity. To assess evidence of epistasis between alleles at two loci (SNP1 and SNP2) a likelihood ratio test (LRT) comparing nested models with and without the interaction was performed. The LRT is based on the likelihood ratio statistic (LRS) with a χ^2 ^distribution with 1 degree of freedom: LRS = 2(L1-L0) where L1 is the maximum log likelihood including an interaction term between SNP1 and SNP2 and L0 is the log likelihood under the null hypothesis of no effect modification of SNP2 on the risk effects of SNP1, L0 = α+β1 (SNP1)+β2 (SNP2). Genotypic interactions were analysed similarly but allowing for the different types of main effect for each genotype (table 1) Values of p = 0.05 were considered statistically significant. P-values have not been adjusted for multiple testing. Analysis was performed using the STATA v8 package.

## Results

### 1 (i) Estimation of risk effects across a 330-kb region of chromosome 4q

**Figure 1:**
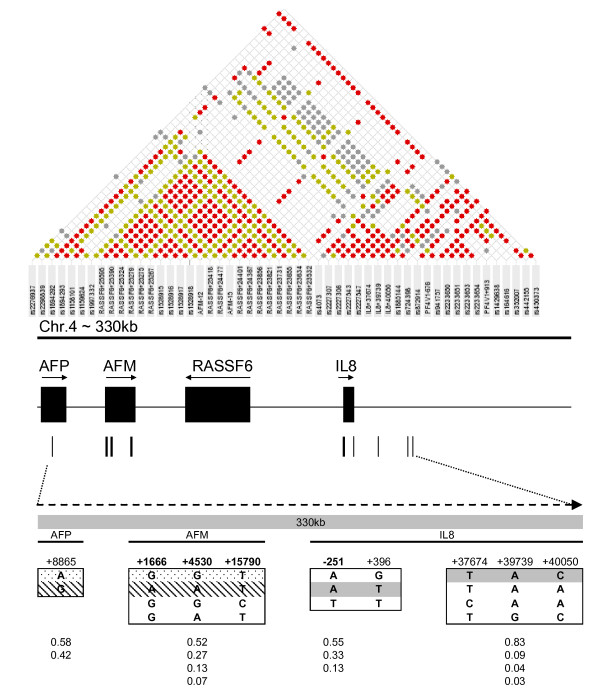
**Top: Hapmap data of LD spanning a 330 kb segment of chromosome 4q in the Yoruba (Nigerian) population (LD map drawn using MARKER (see methods))**; LD between each SNP pair is colour-coded (red dots represent an absolute R^2 ^> 0.3, green dots indicate R^2 ^of 0.1 to 0.3, grey dots indicate R^2 ^of 0.05 to 0.1 and absence of dots represents pairs with R^2 ^≤ 0.05). **Middle**: Genes on the segment are indicated as black boxes, the position of the 9 SNP markers as vertical lines below the boxes, with arrows indicating direction of transcription. **Bottom**: Haplotype structure of the genomic segment in Gambians illustrating four haplotype blocks of high within- and low between-cluster diversity and the haplotype frequencies within each block beneath. Dots and shading indicate the configuration of the protective and risk haplotypes respectively. The dark shading spanning the IL8 locus shows the configuration in blocks 3 and 4 which is shared by the risk and protective haplotypes, differing only at SNP positions 1, 2 and 3 across the AFP and AFM loci but sharing allelic configuration at sites within, and telomeric to, IL8.

DNA from 373 Gambian case-control pairs was genotyped at 9 SNP sites across 330 kb in chromosome 4q (table 1, Figure 1). Full-length population haplotypes inferred using PHASE v2 and population haplotype frequencies determined among cases and controls are shown in table 2. Pair-wise comparisons of R^2 ^between the 9 typed markers are similar to those identified in the Yoruba (Figure 1) and Caucasian populations [20,21] with two broad clusters of association of high-to-moderate pair-wise LD (R^2 ^> 0.1); cluster 1 including AFP and AFM genes and cluster 2 including IL8 and its downstream flanking region (data not shown).

Three out of 11 inferred haplotypes were present at population frequency ≥10% and accounted for 60% of all the haplotypes present in the population (table 2), suggesting a low haplotype diversity in this genomic region in Gambians. The haplotype AFP+8865G/AFM+1666A,/AFM+4530A/AFM+15790T/IL8-251A/IL8+396T/IL8+37674T/IL8+39739A/IL8+40050C, or GAATATTAC, was associated with increased risk of scarring trachoma, at borderline of statistical significance, and the haplotype AGGTATTAC was associated with significant protection from disease (table 2). In CLR analysis the risk of scarring increased with copy number of the GAATATTAC haplotype and it decreased with increasing copy number of the AGGTATTAC haplotype (table 3). The risk effects detected by the high risk haplotype were not affected by those observed for the low risk haplotype (and *vice versa*) (data not shown), suggesting that at least two independent risk effects may operate in the region.

**Table 3:** risk effects estimated by OR (95%CI) for the association between extended (H1, L1) and reduced (H2, L2) haplotypes with scarring trachoma and trichiasis.

HAP^a^	**SNP **^b^									CLR test for trend					
										Scarring trachoma			Trichiasis		
	*1*	*2*	*3*	*4*	*5*	*6*	*7*	*8*	*9*	*OR*	*95%CI*	*p value*	*OR*	*95%CI*	*p value*
**H1**	G	A	A	T	A	T	T	A	C	1.671	1.19,2.35	0.003	1.616	1.08, 2.42	0.020
**H2**	-	**A**	**A**	**-**	**A**	-	-	-	-	1.541	1.14,2.08	0.005	1.510	1.05, 2.15	0.025
**L1**	A	G	G	T	A	T	T	A	C	0.556	0.36,0.87	0.010	0.685	0.40, 1.16	0.161
**L2**	-	**G**	**G**	**-**	**A**	-	-	-	-	0.490	0.27,0.89	0.021	0.725	0.378,1.39	0.334

^a ^Significant associations are fine-mapped to four contiguous ht SNPs bounded by AFM+1666 and IL8-251 (H2 and L2 haplotypes). Regression modelling with PHASE inferred haplotypes from progressively reducing SNP sets showed increased risk effects of similar magnitude in the reduced set compared to those detected by the full length haplotypes.

^b ^Refer to table 1 for SNP information

Pair-wise measures of linkage disequilibrium and regression modelling (figure 1, table 3) mapped the risk associations to the four contiguous SNPs bounded by AFM+1666 and IL8-251 (AFM+1666A/AFM+4530A/AFM+15790T/IL8-251A OR(95%CI) = 2.00 (1.30, 2,94) and 1.610 (0.99, 2.61) for scarring and trichiasis respectively). CLR analyses suggested that the AFM+1666 and AFM+4530 SNP sites explain mainly the high risk effects detected by the risk AATA (H2) haplotype whereas IL8-251 SNP tags some of the risk effects captured by the GGTA (L2) haplotype (data not shown).

When the risk haplotype tagging SNPs AFM+1666, AFM+4530, AFM+15790 and IL8-251 were retested for disease association in the complete case-control sample set of 651 case-control pairs, the AATA haplotype, present in 23% of the population, was found to be associated with an increased risk of trichiasis (OR = 1.39, 95%CI = 1.03, 1.88) with risk effects increasing 40% for each copy of the haplotype (OR = 1.40, 95%CI = 1.04, 1.87). On the other hand the rare IL8-251TT genotype (2.4% population frequency) was associated with reduced risk of scarring trachoma (OR for scarring 0.29, 95% CI = 0.09, 0.87, p = 0.027 and for trichiasis 0.50, 95%CI = 0.04, 5.51, p = 0.571 for IL8-251 TT versus IL8-251 AA+AT). The relative risk of developing scarring among IL8-251 TT homozygotes was estimated to be 2 times lower in the younger group (<35 years old) than in the older group (≥ 35 years), although this difference in risks was not statistically significant possibly due to small sample sizes (data not shown). The risk effects of the AATA haplotype were independent of the IL8-251 TT protective effects suggesting that the risk tagged by the haplotype may be independent of that detected by the IL8-251 TT genotype (data not shown).

### 1 (ii) Epistasis between IL8-251 and MMP9 Q279R SNPs

There was some evidence for interaction between the MMP9 Q279R and IL8-251 alleles affecting risk of scarring trachoma (LRT χ^2 ^= 4.14, p = 0.042). The protective effect of the IL8 T allele was significantly increased, by more than 70%, in subjects carrying the MMP9 G protective allele (OR, 95%CI = 0.352 (0.14, 0.87) but suppressed in the presence of the MMP9 Q279R A risk allele (OR, (95%CI) = 1.90, (0.67, 1.21). No significant interaction effects were noted at the genotype level, although the number of IL8-251TT homozygotes is small. There was no evidence for gene-gene interaction involving MMP9 Q279R and either AFM+1666 or AFM+4500 (data not shown).

### 2(i) Estimation of risk effects across a 656 kb region of chromosome 5q

**Figure 2:**
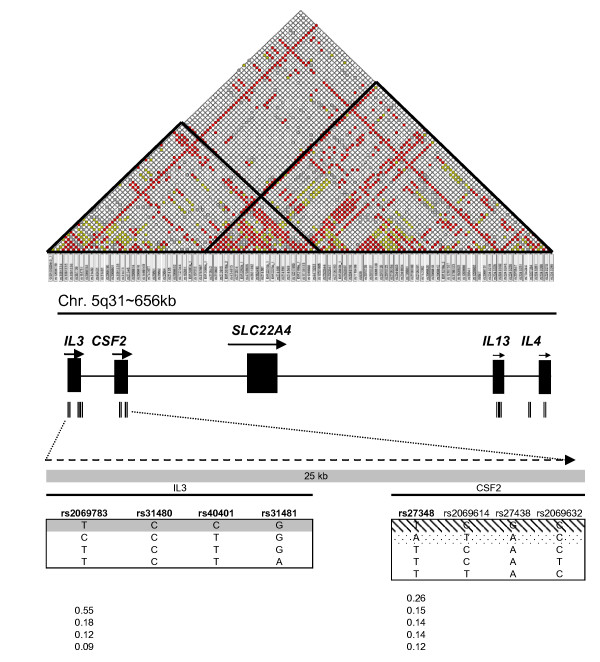
**Top: LD map across a 656 kb segment of the 5q31 region in the Gambian population **[22]. **Middle**: Distribution of the 16 most informative markers (Table 4) that capture the haplotype block structure extending from IL3 to IL4. **Bottom**; Haplotype structure defined by 8 SNP markers across IL3 and CSF2 illustrating two haplotype blocks and haplotype frequencies within each block beneath. Dots and shading indicate the configuration of the protective and risk haplotypes respectively; the dark shading spanning the IL3 locus shows the haplotype configuration shared by the risk and protective haplotypes.

In brief, due to the irregular LD between the IL3/CSF2 and IL13/IL4 regions (Figure 2), genotyping data were used to construct 8 marker haplotypes across each of these two regions in 746 unrelated chromosome pairs (Table 4). LD statistics obtained from the haplotype data were in agreement with published data [22]. A significant association was observed between two 8 SNP-haplotypes delimiting a genomic block of high LD across IL3 and CSF2, and risk of scarring (Figure 2; tables 5 and 6). No significant risk effects were detected across the IL13 and IL4 loci (data not shown).

**Table 4:** list of haplotype tagging SNPs on chromosome 5q genotyped in 373 case-control pairs.

SNP position in haplotype A or B	SNP id^a^	Chromosome position	Database id	SNP genomic location	COMMON allele (minor allele)	Allele frequency
1A	IL3_2069783	131471923	rs2069783	5'UTR	T(c)	0.18
2A	IL3_31480	131472548	rs31480	5'UTR	G(a)	0.05
3A	IL3_40401	131472694	rs40401	exon	C(t)	0.45
4A	IL3_31481	131473418	rs31481	intron	G(a)	0.14
5A	**CSF2_27348**	131483355	rs27348	3'UTR	T(a)	0.39
6A	**CSF2_2069614**	131483817	rs2069614	3'UTR	T(c)	0.45
7A	**CSF2_27438**	131489471	rs27438	3'UTR	A(g)	0.23
8A	**CSF2_2069632**	131489516	rs2069632	3'UTR	C(t)	0.27
1B	IL13_46457	132072180	rs20541	exon	C(t)	0.14
2B	IL13_46578	132072059	rs1295686	splice site	A(g)	0.27
3B	IL13_49612	132069025	rs1800925	5'UTR	G(a)	0.40
4B	IL4-589	132085370	rs2243250	5'UTR	T(c)	0.27
5B	IL4+33	132085926	rs2070874	exon	C(t)	0.49
6B	IL4_2243251	132086003	rs2243251	5'UTR	T(c)	0.19
7B	IL4_2227284	132088941	rs2227284	intron	A(c)	0.05
8B	IL4_2243270	132090325	rs2243270	intron	C(t)	0.27

SNPs given in bold were selected for typing in the complete sample set of 651 case-control pairs

^a^SNPs given in bold were selected for typing in the complete sample set of 651 case-control pairs.

**Table 5:** Risk effects estimated by OR (95%CI) for the association between extended and reduced haplotypes across IL3 and CSF2 and risk of scarring trachoma and trichiasis.

HAP	SNP^a^								CLR					
									Scarring trachoma			Trichiasis		
	*1A*	*2A*	*3A*	*4A*	*5A*	*6A*	*7A*	*8A*	*OR*	*95%CI*	*p value*	*OR*	*95%CI*	*p value*
**H1**	T	C	C	G	T	C	G	C	1.370	1.070,1.763	0.010	1.390	1.040,1.869	0.030
**H2**	-	-	-	-	**T**	**C**	**G**	-	1.032	0.830,1.280	0.778	1.131	0.822,1.556	0.448
**L1**	T	C	C	G	A	T	A	C	0.804	0.520,1.220	0.324	0.871	0.520,1.378	0.609
**L2**	-	-	-	-	**A**	**T**	**A**	-	0.820	0.650,1.043	0.084	0.610	0.423,0.880	0.008

^a ^Refer to table 4 for SNP information

**Table 6:** Test for trend in risk of scarring trachoma or trichiasis with copy number for each haplotype.

HAP	SNP^a^								CLR test for trend					
									Scarring trachoma			Trichiasis		
	*1A*	*2A*	*3A*	*4A*	*5A*	*6A*	*7A*	*8A*	*OR*	*95%CI*	*p value*	*OR*	*95%CI*	*p value*
**H1**	T	C	C	G	T	C	G	C	1.300	1.002,1.670	0.048	1.322	0.980,1.790	0.071
**H2**	-	-	-	-	**T**	**C**	**G**	-	1.086	0.854,1.380	0.502	1.242	0.935,1.645	0.135
**L1**	T	C	C	G	A	T	A	C	0.839	0.555,1.267	0.403	0.656	0.405,1.060	0.087
**L2**	-	-	-	-	**A**	**T**	**A**	-	0.755	0.567,1.005	0.065	0.617	0.438,0.870	0.006

^OR >1 indicates an increase in risk with copy number and OR <1 a decrease.^

^a^Refer to table 4 for SNP information

Following a similar approach to that of the IL8 region, PHASE assignments on progressively reducing SNP sets fine-mapped SNP-haplotype risk effects to 3 contiguous haplotype tagging SNPs bounded by CSF2_27348 and CSF2_27438 spanning the IL3 and CSF2 loci, (figure 2, tables 5 and 6). The high and low risk extended haplotypes shared the SNP alleles in IL3 (tables 5 and 6).

The CSF2_27348A/CSF2_2069614T/CSF2_27438A haplotype (ATA), of 17% population frequency, was found to be associated with lower risk of scarring and trichiasis (OR, (95%CI) of 0.82, (0.65, 1.04) and 0.61, (0.42, 1.88) for scarring trachoma and trichiasis respectively), decreasing with copy number (OR, 95%CI for test for trend of 0.82(0.66, 1.01) and OR of 0.65 (0.47, 0.89) for scarring trachoma and trichiasis respectively). Among common population haplotypes, ATA is unique in that it exclusively carries the CSF2_27348 A low risk allele. The risk effects detected by haplotype and single-marker analyses are very close (tables 5, 6, 7 &8) adding evidence to the idea that ATA haplotype and the CSF2_27348 A allele may capture the same risk effects within the region. This was further confirmed by logistic regression analyses showing that the haplotype risk effects are not significant when adjusted for the risk effects of CSF2_27348 (and *vice versa*) (data not shown). The risk associated with the CSF2_27438 SNP is best described by a recessive model (GG vs AG + AA genotypes). A protective effect at CSF2_27348 was also most marked in a recessive model for the minor genotype AA vs TT+AT. There was some evidence for a dose-response effect towards decreasing risk of disease with increasing number of copies of the CSF2_27348 A minor allele (tables 7 and 8).

**Table 7:** Proportions of cases and controls with different SNP-genotypes at the CSF2 locus and risk estimates from conditional logistic regression (CLR) analysis of 651 matched case-control pairs for each SNP for scarring trachoma.

			Multiplicative model		Dominant model	Recessive model
Genotype	# of genotypes (freq)		CLR		OR (95%CI)	OR (95%CI)
	TS cases	controls	OR (95%CI)	P value	P value	P value
GM-CSF2_27348 TT	391(0.65)	384(0.61)	Reference	-	0.86(0.67,1.09)	0.60(0.33,0.084)
GM-CSF2_27348 TA	193(0.32)	207(0.33)	0.90(0.70,1.57)	0.414	0.200	0.084
GM-CSF2_27348 AA	22 (0.04)	36 (0.06)	0.58(0.32,1.04)	0.069		
**Test for trend**			**0.84(0.69,1.03)**	**0.091**		
GM-CSF2_2069614 CC	206(0.32)	198(0.31)	Reference	-	0.88(0.68,1.28)	0.99 (0.74,1.31)
GM-CSF2_2069614 CT	306(0.48)	316(0.49)	0.87(0.67,1.31)	0.300	0.310	0.922
GM-CSF2_2069614 TT	124(0.19)	130(0.20)	0.90(0.65,1.26)	0.546		
**Test for trend**			**0.94(0.80,1.11)**	**0.467**		
GM-CSF2_27438 AA	215(0.33)	233(0.36)	Reference	-	1.08(0.85,1.37)	1.37 (1.02,1.85)
GM-CSF2_27438 AG	301(0.47)	318(0.49)	1.00(0.78,1.29)	0.991	0.542	0.037
GM-CSF2_27438 GG	126(0.20)	100(0.15)	1.37(0.98,1.93)	0.066		
**Test for trend**			**1.14(0.97,1.35)**	**0.113**		

**Table 8:** Proportions of cases and controls with different SNP-genotypes at the CSF2 locus and risk estimates from conditional logistic regression (CLR) analysis of matched 307 case-control pairs for each SNP for trichiasis

			Multiplicative model		Dominant model	Recessive model
Genotype	# of genotypes (freq)		CLR		OR (95%CI)	OR (95%CI)
	TT cases	controls	OR (95%CI)	P value	P value	P value
GM-CSF2_27348 TT	198(0.69)	179(0.59)	Reference	-	0.62(0.42,0.90)	0.42 (0.18,0.98)
GM-CSF2_27348 TA	82 (0.28)	103(0.34)	0.67(0.45,0.99)	0.048	0.012	0.046
GM-CSF2_27348 AA	9 (0.03)	20 (0.07)	0.37(0.16,0.87)	0.024		
**Test for trend**			**0.64(0.47,0.88)**	**0.005**		
GM-CSF2_2069614CC	108(0.36)	94 (0.31)	Reference	-	0.77(0.57,1.12)	0.82 (0.53,1.26)
GM-CSF2_2069614 CT	138(0.46)	146(0.48)	0.79(0.53,1.18)	0.254	0.171	0.359
GM-CSF2_2069614 TT	54 (0.18)	65 (0.21)	0.70(0.42,1.16)	0.172		
**Test for trend**			**0.83(0.65,1.07)**	**0.154**		
GM-CSF2_27438 AA	101(0.33)	127(0.40)	Reference	-	1.29(0.91,1.83)	1.14 (0.74,1.75)
GM-CSF2_27438 AG	145(0.48)	132(0.42)	1.28(0.88,1.85)	0.195	0.157	0.56
GM-CSF2_27438 GG	57 (0.19)	55(0.18)	1.31(0.81,2.13)	0.268		
**Test for trend**			**1.17(0.92,1.48)**	**0.203**		

### 2 (ii) Epistatic effects between CSF2_27348 and MMP9 Q279R SNPs

Evidence for interaction was observed between genotypes at the MMP9 Q279R and CSF2_27348 loci (CSF2_27348 - MMP9 Q279R recessive-heterozygote advantage disease model (LTR χ^2 ^= 8.08 p = 0.005)). MMP9 Q279R AG heterozygotes were most protected from risk of trichiasis when this genotype was combined with the CSF2_27348 TT genotype (OR, (95%CI) = 0.41 (0.25, 0.66). In contrast MMP9 Q279AG/CSF2_27348 AA conferred increased risk of trichiasis (OR, (95%CI) = 7.55 (1.01, 56.71) with wide confidence intervals resulting from the small number of subjects with the CSF2_27348 AA genotype.

## Discussion

By means of combining LD mapping, case-control and epistatic analyses we have identified a genetic association between severe outcome of ocular *Ct *infection and the pro-inflammatory genes IL8 and CSF2 within the chemokine and cytokine gene clusters in chromosomes 4q and 5q31 respectively. These two pro-inflammatory mediators characterise an early host response to *Ct *infection thought to act to augment the innate immunity essential for host defence or, if unchecked, cause cytotoxic damage to the epithelium [14,15,26].

Homozygotes for the IL8-251T promoter region allele showed a greatly reduced risk of scarring trachoma. However the prevalence of the IL8-251TT genotype was low (2.4%) in our population indicating that a larger case-control series will be needed to get accurate estimates of risk effects. The degree of protection was greater in younger compared to older age groups suggesting that effects linked to IL8-251 may be particularly relevant to the development of scarring trachoma and trichiasis at an early age. Cases and controls were matched by age and village of residence, so that this age difference is not accounted for by different treatment history (treatment is programmed per village) or environmental influences. The IL-251A common allele has been correlated with high IL8 expression *in vitro *[27] and also with an increased susceptibility to respiratory syncytial virus (RSV) bronchiolitis [21,27], a chronic inflammatory disease characterised by high levels of IL8 and neutrophil infiltration of the airway epithelium. It is therefore plausible that, in IL8-251T homozygotes, genetically controlled production of low IL-8 levels by *Ct *infected cells in the conjunctival epithelium leads to decreased activation and migration of neutrophils to the site of infection. The subsequent reduction in inflammation and cytotoxic epithelial damage, may ultimately protect the conjunctiva from the development of severe trachoma sequelae. The genetic risk effects for scarring and trichiasis observed in this genomic region are similar, which suggests that the above molecular mechanism may be common to the development of both phenotypes, rather than implicated in the progression of scarring to trichiasis.

Fine mapping of susceptibility to RSV bronchiolitis at this locus [21] suggested that additional risk variants in the region mapped to a region excluding the AFM gene (which codes for an albumin-like protein expressed in the liver) but including the novel gene RASSF6, a member of the Ras superfamily of small GTPases that can mediate bacterial entry by controlling changes in the actin cytoskeleton [28-30]. Here we show that the risk effect on trachoma maps to the region bounded by the AFM+1666 and IL-251 markers which contains RASSF6 (Figure 1). Interestingly, by means of gene expression microarray analyses, we have found that the RASSF6 expression pattern maps to a cluster of genes involved in reorganization of the actin cytoskeleton whose expression is up-regulated in the *Ct *infected conjunctiva of Gambians (unpublished data).

The functionality of the 2 intronic risk variants detected here across CSF2 is not obvious from genome annotation and it is possible that these SNPs act only as markers for a yet unidentified functional element. The CSF2_27348 marker has been found to be linked to the rs721121 SNP, located in an enhancer site for the inducible transcription factor AP1, an intergenic enhancer required for the correctly regulated activation of both CSF2 and IL3 gene expression in T cells [31]. Among other genes neighbouring CSF2 that need to be considered as candidates for the origin of the risk effects is SLC22A4 (Figure 2). This gene codes for an organic cation transported in lymphoid organs [32] which has been associated with risk of rheumatoid arthritis [32]. We have recently found that the transcriptional levels of SLC22A4 are increased in the conjunctiva of subjects with inflammatory trachoma and ocular *Ct *infection, mapping to a gene cluster characteristic of an innate immune response pattern of expression (unpublished data). Similarly to RASSF6, the role of SLC22A4 in the inflamed conjunctiva remains to be elucidated.

The statistical interaction (epistasis) observed between the risk variants within IL8 and CSF2 and the Q279R exonic SNP in MMP9, a collagenase thought to be involved in the inflammatory and scarring processes of *Ct *infection [11,33,34], may further suggest how genetic variation detected in IL8 and CSF2 could affect risk of trachoma: IL8, CSF2 and MMP9 are co-expressed in the *Ct *infected conjunctiva [34], unpublished data). These gene products could interact at the site of infection to augment and sustain inflammatory processes. MMP9 has been found to greatly enhance the activity of IL8 by amino-terminal processing[35], whereas activation of neutrophils by IL-8 may trigger the release of pro-MMP9 [36,37], creating a potential for a positive feedback loop. Similarly, CSF2 release by *Ct *infected epithelial cells [15,16] may mediate the influx and activation of inflammatory cells at the site of infection. Secreted CSF2 may trigger MMP9 production by monocytes [38], which could act to enhance and sustain the pro-inflammatory cascade initiated by CSF2. Thus the epistatic genetic risk effects could reflect biological interactions between their products, allowing a mechanism by which genetic variation affecting IL8 and CSF2 expression/activity change the risk effects associated with MMP9 variants and *vice versa*. Epistasis has not been reported before in studies of susceptibility to trachoma but may be an important feature of highly regulated gene networks.

## Conclusion

This study provides the first evidence that genetic susceptibility determinants for severe sequelae of ocular *Ct *infection may exist among innate response genes. This work adds to recent observations [39,40] suggesting that innate immune responses in early inflammatory events during acute infection with *Ct *may have both favourable and detrimental effects in the development of irreversible sequelae of *Ct *infection. Although we narrow down the limits of disease association to a few candidates within these genomic regions, which are complex and rich in immune genes, the results should be interpreted with caution. Functionally important SNPs directly influencing risk of disease remain to be uncovered; the complex and locus-specific architecture of the 4q and 5q31 genomic regions requires 'deeper' genotyping with a higher density of markers and increased sample sizes.

## Competing interests

The authors declare that they have no competing interests.

## Authors' contributions

AN collected, edited, analysed the data and wrote the manuscript. RB directed the study, participated in study design and co-wrote the manuscript. JH and GL participated in study design. DM, MH, MB, KR and DK co-directed the study, participated in study design and contributed to the manuscript. HJ collected clinical data and provide clinical material.

## Pre-publication history

The pre-publication history for this paper can be accessed here:

http://www.biomedcentral.com/1471-2350/10/138/prepub
